# Social Navigation in a Cognitive Architecture Using Dynamic Proxemic Zones

**DOI:** 10.3390/s19235189

**Published:** 2019-11-27

**Authors:** Jonatan Ginés, Francisco Martín, David Vargas, Francisco J. Rodríguez, Vicente Matellán

**Affiliations:** 1 Intelligent Robotics Lab, Rey Juan Carlos University, Campus de Fuenlabrada, Camino del Molino s/n, 28943 Fuenlabrada, Spain; francisco.rico@urjc.es (F.M.); d.vargas@alumnos.urjc.es (D.V.); 2 Robotics Group, University of León, Campus de Vegazana, s/n, 24071 León, Spain; fjrodl@unileon.es (F.J.R.); vicente.matellan@unileon.es (V.M.)

**Keywords:** proxemics, social navigation, social robotics, mobile robots

## Abstract

Robots have begun to populate the everyday environments of human beings. These social robots must perform their tasks without disturbing the people with whom they share their environment. This paper proposes a navigation algorithm for robots that is acceptable to people. Robots will detect the personal areas of humans, to carry out their tasks, generating navigation routes that have less impact on human activities. The main novelty of this work is that the robot will perceive the moods of people to adjust the size of proxemic areas. This work will contribute to making the presence of robots in human-populated environments more acceptable. As a result, we have integrated this approach into a cognitive architecture designed to perform tasks in human-populated environments. The paper provides quantitative experimental results in two scenarios: controlled, including social navigation metrics in comparison with a traditional navigation method, and non-controlled, in robotic competitions where different studies of social robotics are measured.

## 1. Introduction

We focus on social robots that operate in human-populated environments and can interact with them. Although it is not yet common to live with a robot every day, this will soon be a reality. However, there is still much to do to make these robots acceptable in our daily life.

In mobile robotics, the main objective of a navigation system is to allow a robot to move from one point to another in the environment safely and efficiently. In this work, we focus on cases where the environment is known a priori, and coded on a map. Having a reliable and stable location is critical to the success of navigation. Most of the successful navigation approaches divide the navigation modules into two levels: global planner and local planner.

The global planner calculates the route from the robot’s current position to the final position. We can use multiple algorithms for this task: A* [1], Dijkstra algorithm [2], Gradient Methods [3], etc. In our work, we used an A* algorithm for calculating routes on a cost map. A cost map encodes the obstacles of an environment on a grid. There is a value that indicates that a grid cell is unknown, a maximum value that indicates that there is an obstacle, and a minimum value that indicates that it is clear. Intermediate values indicate the cost of going through this grid cell. They do not block but involve a higher cost when calculating the route.

The local planner is responsible for following the routes generated by the global planner generating movement commands. It also has to avoid possible obstacles that are present on the map. Among the algorithms used in these planners are Dynamic Window Approach (DWA) [4], Elastic Bands [5], Timed-Elastic-Band [6,7], Trajectory Rollout [8], Genetic approaches [9,10], and mixed approaches [11,12,13].

We developed [14] one level above to plan the movement of a robot through indoor environments. We call it *topological navigation*. A robot may have to go through doors, climb elevators, or pass-through access lathes. We used classical PDDL-based [15] planning to divide navigation into actions. These actions can be simple (move_in_a_room and move_to_accessible_room), which only require navigation, or more complex (open_door and call_elevator), which may require manipulation or dialogue.

We integrate this topological navigation module in our cognitive architecture. Our architecture allows developing software modules, such as the case of topological navigation, which provides portions of PDDL to the application domain. Each module provides the implementation of each of the actions, such as the actions described above. In addition, each module implements a set of skills (perceptual or acting) that may be required to carry out the actions.

The challenges we face in social robotics are numerous. A robot is not perceived in the same way by all humans. There are people more likely to interact with robots. Furthermore, some people are bothered to be near a robot. A robot should respect people’s privacy, not accessing places where it would be annoying for a person to find a robot (in a bedroom or a bathroom). A robot should not cross areas of high activity of people, because it could be a hindrance (a kitchen, for example). A robot should not trace routes through groups of people who are interacting, or areas that limit people’s activities (a robot should not pass in front of the television if people are watching it, for example). If a robot takes these factors into account when navigating, it will contribute to making people accept the robot in living with people.

We consider that social navigation is also a robot skill at a higher level than a global planner. A social robot must trace its routes using a map, but also with certain restrictions. The motivation is that the robot disturbs people in the environment as little as possible while navigating. It should also not pass near persons whose attitude towards the robot is not favorable. We develope in this paper a social navigation module that inserts restrictions to the local navigation planner. We carry out this duty by modifying the costs of the map cells used by the local planner. These restrictions are made based on the mood of the people detected by the robot.

We develope our social navigation module as a module within our cognitive architecture. We mainly implement the social_move action. To carry out this action, we develop several perceptual skills to detect people, their intentions, and their humor, among other characteristics.

In summary, the main contributions of our work in this paper are:

- We model the area of influence of people based on the perception of their attitude towards the robot. We code these areas in cost maps.
- We integrate a social navigation behavior in a cognitive architecture.
- We test our approach in a competition environment.

This paper is structured as follows: In Section 2, we present all the works we consider relevant in the area of social robotics. In Section 3, we briefly describe the cognitive architecture in which our social navigation system is integrated. In Section 4, we present the complete social navigation system, which is the main contribution of this work. In Section 5, we show experiments that validate our approach. Finally, Section 6 contains the conclusions of this work.

## 2. Related Work

Social robotics is the area that studies the use of robotics technologies in a domestic environment and research on better ways for the robots to adapt to daily human lives [16]. Within this area is the social navigation, a field of knowledge that works on improving the movements of a robot when it is in a populated environment. This field has attracted broad interest in the last years [17,18,19]. The primary motivation of social navigation is to disturb people as little as possible, in addition to following certain social conventions that will be determined by culture, age, or context. The first works in this area propose a virtual force model or social force model [20], in which authors model the forces acting on a person that is walking in a crowded scenario as a combination of three forces, one to describe the acceleration, another repulsive force to describe the distance to other pedestrian and borders, and the last one to describe the attractive effects of the pedestrian target. A recent work in this line is in [21], in which the authors used Social Force Model (SFM) to avoid humans in an urban scenario.

The use of emotions also could be significant in social navigation. The authors of [22,23] established that emotions, particularly fear, influence the size of our personal space. Taking this into account, robot navigation must be modified to ensure that they will be accepted by a society in which many people are afraid or distrustful of robots. Another use of emotions in robotics is in [24], where the authors proposed that a robot could change its navigation mode according to a collection of different emotions that the robot could perform.

There are also learning-based methods (e.g., [25,26,27]), in which the robot learns from the human behaviors when a person navigates in a populate scenario. It generates a set of parameters that are used afterwards for planning or following a path in a socially accepted way. Tranberg Hansen et al. [28] used a learn-based method to estimate if a person wants to interact with the robot. If not, the robot must respect the personal space of the person and go out if it is invading it.

Other works propose the use of fuzzy logic [29] to achieve that a robot could accompany a person for a corridor, avoiding cross in front of moving people, or the use of the proxemics theory [30] to solve the problem of a robot stands in line with humans.

Proxemics theory is the basis of a large number of studies, mainly for its social and human-centered focus. It defines the space around a person as different zones with different radii: intimate, personal, social, and public. Some works use the proxemic zones to feed the global planner [31,32], creating a social path to adapt its behavior to people and the environment. Our social navigation method, conversely, modifies the local cost map achieving a reactive behavior and allowing that the global planner could replan.

The proxemics theory does not describe areas with a fixed radius but defines areas that could change according to the culture or age, among others. Some works address the problem of walking in a corridor using proxemics theory as a base [33,34]. These works develop methods to follow the social convention of keep on the right when walking in a corridor. However, our proposal is general to any scenario.

In [35], the authors proposed an approach where the proxemic zones are dynamic and change depending on the spatial context and human intention.

This paper focuses on improving the comfort [17] of people creating dynamic proxemic zones that are modified by different parameters, i.e., attitude towards the robot, age, or presenting behavior that indicates the intention of interact with it. Furthermore, the proxemics theory is given a similar approach to Tranberg Hansen et al. [28], who used proxemic zones, not only for creating a forbidden navigate zone for robots but also under certain conditions that allow human–robot interaction.

## 3. A Cognitive Architecture for Social Robots

**Figure 1:**
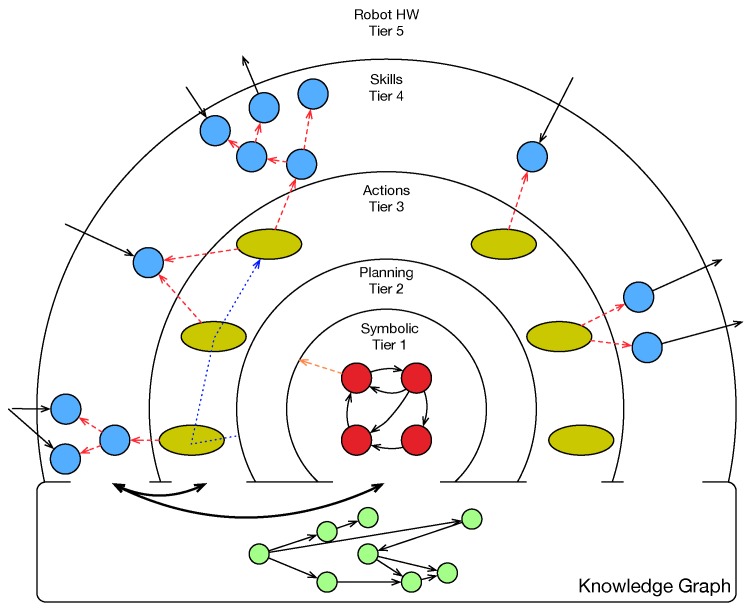
Layered cognitive architecture.

Figure 1 shows the design on layers of our architecture. It is a concentric-layered design, with a transversal component called *knowledge graph*. The knowledge graph stores the internal and external knowledge of the robot, and it is accessible from any layer.

Tiers 1 and 2 mainly use symbolic information. Tiers 3 and 4 use subsymbolic information. The central part of the architecture, at Tier 2, is a symbolic planner based on PDDL. Using PDDL, we define what types, symbolic predicates, and actions can be used to solve a problem in a domain. This planner has a knowledge base, accessible from other levels, that contains the instances and predicates of the current problem.

- **Tier 1** states the instances and predicates of the problem to be solved. This level contains hierarchical state machines that define the modes and behaviors of the robot at a high level. We implement transitions between states by consulting predicates in the knowledge base. We define goals for the planner in the states.
- When a state machine at Tier 1 establishes a goal, the planner at **Tier 2** creates a plan using the content of his knowledge base. The plan is made up of a sequence of domain actions. The planner delivers the actions at Tier 3 one at a time. Each time an action indicates that it has been completed successfully, the next one is delivered until the plan is finished. If an action finishes with error, it forces a replanning.
- **Tier 3** contains the implementation of the actions defined in the PDDL domain. This level is the bridge between both paradigms. The planner activates actions according to the generated plan. When activated, the planner passes the parameters to the actions (instances of a type). Usually, the action must translate symbols into specific data. For example, a move action could receive *kitchen* as a parameter. Then, the action must obtain the metric coordinate corresponding to the *kitchen* symbol and send it to the navigation module.
  On some occasions, the actions receive information extracted from the sensors, and send commands to the actuators. Most of the time, the actions are too complicated and delegate part of their operation to skills. The actions activate skills and monitor when they have carried out their work.
- **Tier 4** contains skills that can be activated from actions. The skills can be reused from any of the actions. This level includes perceptual, attention, dialogue, and manipulation modules, among others.
- The **knowledge graph** stores the information relevant to the operation of the robot. We design this shared representation of data to disengage some components of others, especially between different layers. An action in Tier 3 uses the result of computing a skill in Tier 4 by reading it from the knowledge graph. Tier 1 can also use the symbolic information contained in the graph.

The elements of the graph are nodes and arcs. The nodes represent instances of a specific type. The arcs can contain a text, or they can provide a geometric transformation.

**Figure 2:**
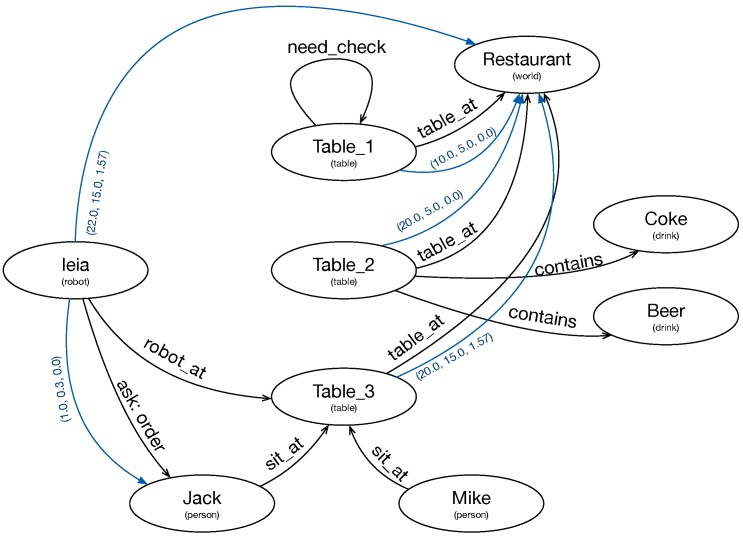
Knowledge graph representing the internal and external knowledge of the robot. Ellipses represent nodes with an ID and a type. Black lines are text arcs and blue lines are geometric arcs.

The relationship between the symbolic information that contains the knowledge base of the Tier 2 planner and the information of the graph is not direct. A process synchronizes the relevant information in the knowledge base of the planner and the graph. We previously configure suitable types and predicates. This process adds nodes to the graph when the knowledge base creates instances of a relevant type. This process also creates arcs when the knowledge base inserts a relevant predicate. If the predicate has two arguments of related types, the arc connects two nodes with a text corresponding to the predicate. If the predicate has only one argument, it is a self arc (*need_check* arc in Figure 2). Currently, the updates only go one way, avoiding updates from the graph to the knowledge base. Figure 2 is the graph of a real application, and all the nodes and arcs (except “ask:”) correspond to instances and predicates from the planner knowledge base.

## 4. Social Navigation

**Figure 3:**
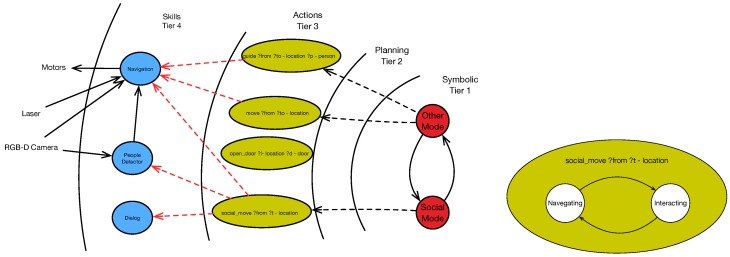
The cognitive architecture (**Left**) and social_move action details (**Right**).

The cognitive architecture just described integrates our navigation system. Figure 3 shows this integration. Before the development of our approach, there were already actions in which the robot had to navigate. The move action moves the robot from one point to another in the environment. The guide action guides a person from one point to another in the environment, using its rear ultrasound sensor to control whether the person follows the robot. The plans that include these actions also include the open_door action in the case of going through doors. Both guide and move use the navigation skill, establishing geometric coordinates as the destination.

We implement our approach through one action and several skills. This new action, social_move, is available to be included in any plan that requires the robot to navigate between people. When this action is activated, it also activates two skills: one to detect people, and another to maintain a dialogue with them, in the case an interaction request occurs. We implement action social_move as a state machine with two states, as shown in Figure 3 (right):

- In the navigating state, this action sends geometric positions to the navigation skill. In this case, we activate a *social layer*, which we detail below.
- In the interacting state, the robot pauses navigation and initiates a dialogue with a person. The transition to this state occurs when a person asks the robot to initiate a dialogue. The robot stops and faces the person. Once the dialogue finishes, the robot continues navigating to the destination.

**Figure 4:**
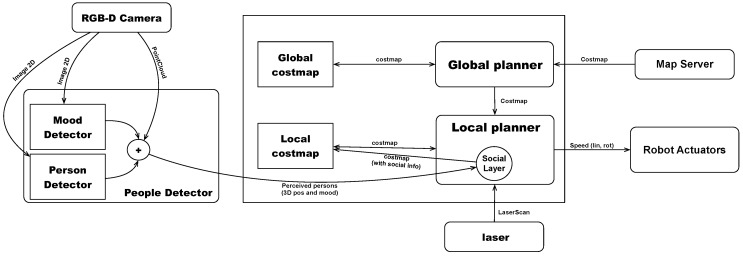
Functional diagram of the proposed approach.

The above description analyzes how we integrate our approach into our cognitive architecture. We implement the core contributions in the people detection skill and the social layer implemented within the navigation skill, shown in Figure 4. Next, we describe in detail the design of both skills.

### 4.1. People Detector Skill

The people detector skill is responsible for detecting the position of people in coordinates of the map reference axis. We represent this skill in Figure 4.

Within this skill, there is a component called person detector, which is responsible for detecting a person within an image using a convolutional neural network. The input of this component is the 2D image of the camera, and the output is a list of detections. Each detection contains a probability and the coordinates, in image coordinates, of the bounding box of the detection.

The mood detector component takes the image as input and generates a list of emotions detected in the faces existing in the image. Each detection contains a mood (positive, neutral, or negative). It also contains the coordinates in the image of this detection.

Both outputs get combined to label the detected people with a mood. If a person is detected and there is no emotion associated with it, we label it as neutral. The detected person is tagged as neutral until this component detects a different mood. This information is persistent in time, and each person is tagged with the last mood that could be detected of him.

**Figure 5:**
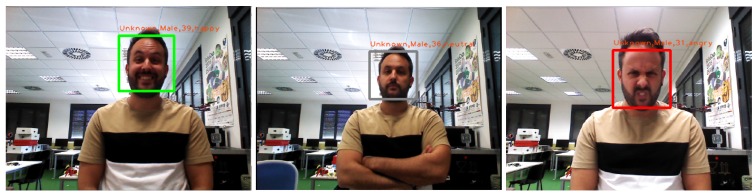
Mood detector detecting different attitudes: (**Left**) positive attitude; (**Central**) neutral; and (**Right**) negative attitude.

Finally, we transform the bounding boxes in coordinates of the image into bounding boxes in three dimensions. To carry out this process, we use the point cloud that corresponds to the image used in the mood detection. The points that make up our point cloud and the pixels of the corresponding 2D image have the same position. An example of this detection is shown in Figure 5.

### 4.2. Navigation Skill

The navigation skill uses the ROS navigation stack [36]. As described above, it consists of two planners, global and local. The global planner calculates the route from robot current position to target, and the local planner generates movements to follow this path, avoiding unexpected obstacles. In addition, this system could be configured by adding new layers [34]. In this paper, we develop a layer, social_layer. It modifies the local cost map adding the proxemic zones related to people.

**Figure 6:**
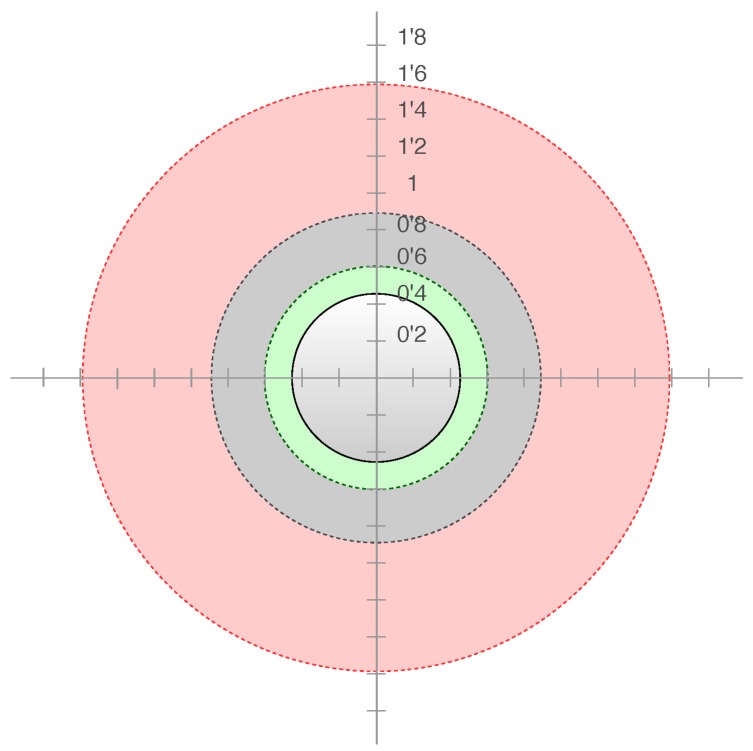
Personal zones details (m): red for a negative attitude, gray for neutral, and green for a positive attitude.

Figure 6 shows a graphic comparison of the different proxemic areas and their size. These zones are coding in the local cost map as Gaussian functions of concentric circles [19,37,38]. The parameter of these Gaussian functions that changes according to the mood of the people is the covariance. In summary, the personal zone radius of a person with a positive attitude towards the robot is 0.6 m, for a person with a neutral attitude is 0.9 m, and for a person with a negative attitude is 1.6 m. The personal zone radius for people with a positive or neutral attitude remains within the limits presented in the literature [19] and, for people with a negative attitude, it is expanded further beyond that limit. In this way, a personal zone is created in line with their negative attitude [22,23] towards the robot, so that their comfort is not affected.

**Figure 7:**
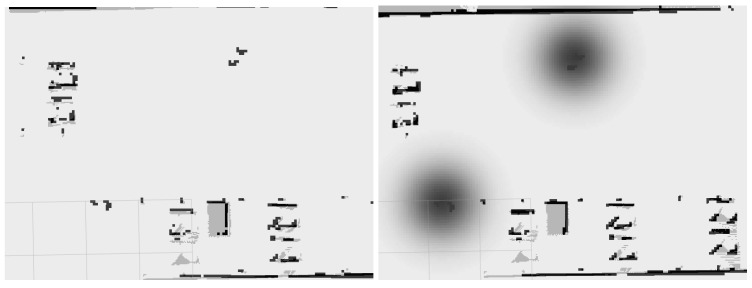
People representation in a local cost map by: default in ROS (**left**); and using proxemic zones (**right**).

With the proxemic zones added in the local cost map (Figure 7), as described in Algorithm 1, and a target position established, the global planner will create a new path to reach the goal. The local planner will generate the correct movements to keep the robot in the cell with less cost, and, because of this, it will keep out of the personal zones of the people.

**Algorithm 1** Proxemic zones to local cost map.
1:
**while** robot_navigates **do**
2:    list People detected_people = get_detect_people_with_mood()3:    **for all** $p_{i}$ ∈ detected_people **do**4:        Float covar = get_covar_from_mood($p_{i}$.mood)5:        Cell person_cell = worldToMap($p_{i}$.position.x, $p_{i}$.position.y)6:        setPersonalZone(local_costmap, person_cell, covar)7:    **end for**8:
**end while**



**Figure 8:**
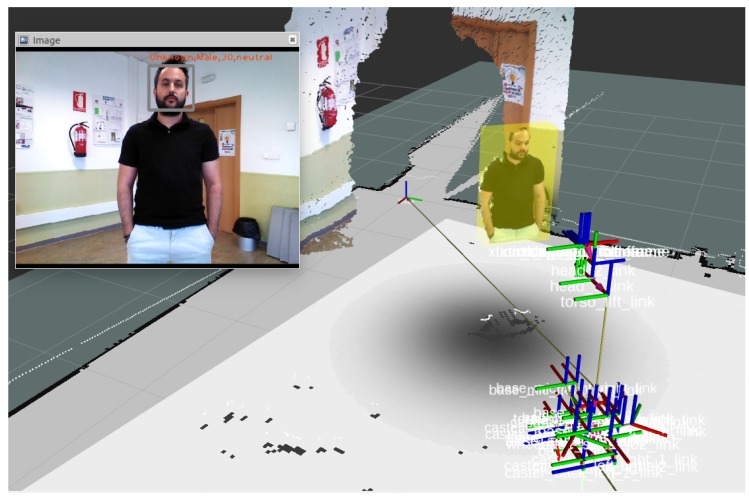
A person detected and positioned in the map with its proxemic zone.

Using this approach, we can reach that the robot does not invade personal zones. Besides, we enable the human–robot interaction if a person has a positive attitude. In the same way, we try not to affect at all a person who is showing a negative attitude towards the robot. Figure 8 shows the integration of the above two skills to create personal zones.

## 5. Experiments

We tested the validity of our approach in two ways. First, we performed experiments in a simulator. Secondly, we tested our approach in a more realistic environment in the SciRoc competition, in which we participated in Milton Keynes in September 2019.

In the implementation of our architecture, we use ROSPlan [39] and BICA [40].

ROSPlan is an IA planning framework, which uses popf [41] among other planners, and is situated in Tier 2 (Figure 1. BICA is a toolbox to create software architectures for robots. Virtually all the elements of our design are BICA components that perform different functions. A BICA component is an independent process that can declare that it depends on other BICA components. When a BICA component is activated, it automatically activates all its dependencies. When all components that enable a dependency are deactivated, the dependence is deactivated. This mechanism is a simple way to save computation time when the results of certain computations are not being used.

Finite state machines in Tier 1 are inside BICA components. As each state can declare a dependency on other BICA components, we can create a hierarchy of state machines. Actions and skills are also BICA components. An action declares some skills as dependencies. When the planner activates an action, all its required skills activate.

### 5.1. Social Navigation in Simulated Scenarios

The tests in simulated scenarios were performed on a computer with an Intel Core i7-8550U 1.8 GHz processor with 16 Gb of DDR4 RAM and Ubuntu GNU/Linux 16.04 using Gazebo as simulator and ROS as robot framework and a Jetson TX2 256-core GPU. YOLO [42,43] was used as the people detector and EmoPy [44] was used as the emotion detector.

We used the metrics already established by the scientific community [45,46,47], formally described in [48], to ascertain the efficacy of the proposed system: $d_{min}$, average minimum distance to a human during navigation; $d_{t}$, distance traveled; τ, navigation time; and Psi, personal space intrusions. The navigation was done at a speed of 0.3 m/s [49].

We compared the proposed algorithm with the ROS traditional navigation since this navigation system is the most widespread and used by the scientific community, and with the static proxemic method described in [34].

**Figure 9:**
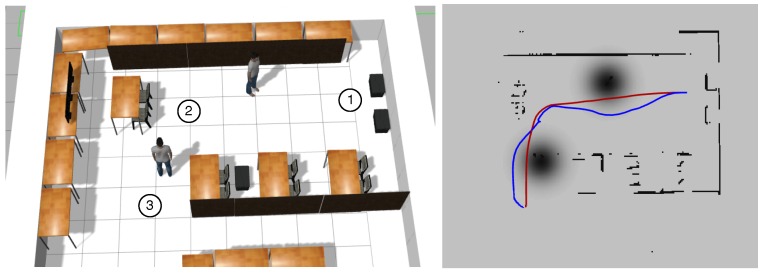
(**Left**) Experiment 1 setup with the reference waypoints. The robot started at Waypoint 1, went to Waypoint 2 and finished at Waypoint 3. (**Right**) Path for ROS traditional navigation in red and the path followed with our approach in blue.

In the first experiment, the robot started at Waypoint 1 (Figure 9), then it navigated to Waypoint 2, and it finished going to Waypoint 3. In this case, the robot did not have information about the mood of the people, so the attitude of everybody was neutral, and the radius of their personal area was 0.9 m. Table 1 shows a comparison between both navigation systems.

It can be seen that $d_{t}$ is slightly higher than the ROS default system, and the distance traveled is longer. This result is justifiable by the social behavior that the robot has with the proposed system. The robot does not invade at any time the personal area of people. Figure 9 shows the path followed by the robot with both systems.

**Figure 10:**
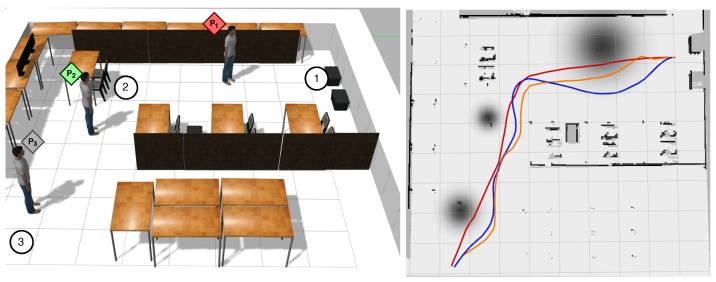
(**Left**) Experiment 2 setup with the reference waypoints and people’s attitude: red for negative attitude, gray for neutral attitude, and green for positive attitude. The robot started at Waypoint 1, went to Waypoint 2, and finished at Waypoint 3. (**Right**) Proxemic zones depending on the people’s mood. The path for ROS traditional navigation is in red, the path for the approach of Lu and Smart [34] is in orange and the path followed with our approach is in blue.

As in Experiment 1, the robot started at Waypoint 1 (Figure 10), it navigated to Waypoint 2, and it finished at Waypoint 3. Figure 10 shows the different moods of people of the scenario, and Table 2 shows the experiment results.

In this case, we can see how the traditional navigation of ROS invades the personal zones of People 1–3, taking into account that Person 1 has a negative attitude towards the robot. Similarly, the approach of Lu and Smart [34] invades the personal zone of Person 1, because it does not take into account the attitude of people. The proposed approach adapts correctly to the environment and does not invade any personal area.

Results from Lu and Smart [34] and our approach are similar. The main difference is produced when a person has a negative attitude. For this reason, we evaluated these methods in a real experiment.

**Figure 11:**
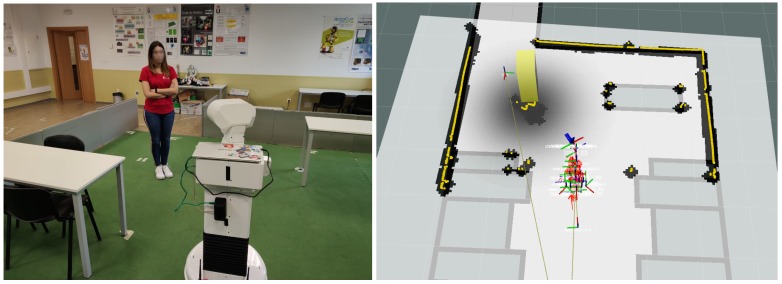
The experiment with real participants to measure their comfort.

The experiment was conducted with real participants to assess their comfort using the approach of Lu and Smart [34] and our approach. Eighteen people aged 18–30 participated (Figure 11). All of them assumed the role of a person with a negative attitude towards the robot, who is afraid or who thinks that the robot could hit them. In each iteration, the robot performed two navigation rounds in which it used each of the approaches. During the route, the robot encountered a person who was obstructing its original trajectory and the robot had to avoid them. After each round, the participant filled out a questionnaire indicating the type of approach used in each case. This information was provided by the authors just before filling in the questionnaire. Then, that participant carried out the second round of the experiment using the approach that had not been used in the previous round. The measurement was a simple rating on a Likert-type scale [50] between 1 (“Completely disagree”) to 5 (“Completely agree”). Table 3 shows the mode [51] of each item of the questionnaire and Table 4 shows the quantitative analysis of these results using the *t*-test method.

In our questionnaire, we asked six questions aimed at the individual’s perception of the robot’s behavior. Although there are no significant differences between the two samples for five of the questions (1, 2, 4, 5, and 6), for Question 3, which is related to the proxemics in HRI scenarios, the results after applying a t-student test (*p* < 0.05, particularly 0.015 in the two-tail sample, Table 4) shows that the application of our approach improves the people’s comfort and how they think that the distance between them and the robot is more appropriate.

### 5.2. Social Navigation in Competition Scenario

**Figure 12:**
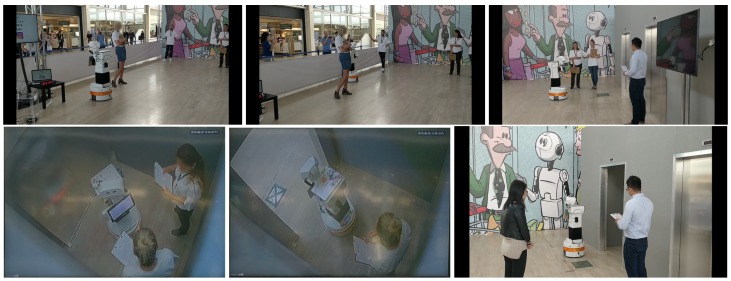
“Take the elevator” challenge of SciRoc 2019.

Robotics competitions are useful because they present a common problem that is addressed by several teams of researchers. In these competitions, metrics are established to measure the robot’s performance, which allows comparing the investigations between the teams. In particular, we participated in the “take the elevator” (https://sciroc.eu/e04-take-the-elevator/) challenge in which the robot must integrate social navigation with other behaviors, Figure 12:

1. The robot must go to an elevator. On his way, he will meet two people. One does not want to interact with the robot, and another person actively attracts the robot’s attention. The robot must maintain a dialogue with this second person.
2. The robot must wait for the elevator to arrive in a position that does not bother the other people who are waiting and allow the people arriving in the elevator to leave.
3. The robot should navigate to a position in an elevator where it does not bother other people, and allow people to get off the elevator on each floor.
4. The robot must exit the elevator when its floor arrives. It can face the people in the elevator to ask for the floor.

The people who judge this test are technical and non-technical volunteers who judge the performance based on a mixed evaluation taking into account both robot performances and a user evaluation that includes, among other things, their comfort when sharing their space with the robot. We, as Gentlebots team and using TIAGo (http://pal-robotics.com/robots/tiago/) robot as robotic platform, won the “take the elevator” challenge (https://sciroc.eu/winners-2019-edition/) in this 2019 edition of SciRoc.

## 6. Conclusions

Nowadays, it is not yet common to live or interact with a robot every day, although the scientific community is working hard to make this a reality soon. Significant efforts are being made so people feel comfortable when a robot shares space with them, and the use of proxemic zones to represent people and their area is the most conventional way.

This work proposes the use of dynamic proxemic zones considering the attitude of people to change their size. In addition, this skill has been integrated into a layered cognitive architecture to be used in conjunction with other capabilities, such as the human–robot interaction system, in different scenarios. Using our approach, the robot behavior when navigating in a social environment is improved. Its performance, compared with a traditional navigation system and a state-of-the-art method, was demonstrated in the experiments.

Talking about the competition, in this challenge, participated robots such as Pepper (https://www.softbankrobotics.com/emea/en/pepper), a more attractive and social robot in aspect than TIAGo. Our success is based on that; not only is the appearance essential in social robotics, but the behaviors also play a fundamental role.

Future works include the improvement of the social layer to consider the age or the intention to interact with the robot. In addition, the ability to recognize different types of humans could be developed, such as older people with a cane, walker, or wheelchair to create proxemic zones with different sizes.

## Figures and Tables

**Table 1:** Social navigation metrics for Experiment 1: for each parameter its mean and standard deviation are provided in parenthesis.The bold numbers indicate a better result in comparison with the other approaches.

Parameter	Proposed Approach	ROS Traditional Navigation
$d_{t}$ (m)	47 (2.65)	**38.67 (2.52)**
τ (s)	8.96 (0.58)	**8.84 (0.23)**
$d_{min}Person1$ (m)	**1.19 (0.14)**	0.57 (0.02)
$d_{min}Person2$ (m)	**1.05 (0.07)**	0.51 (0.03)
Psi(Personal) (%)	**0 (0)**	15.57 (0.39)

**Table 2:** Social navigation metrics for Experiment 2: for each parameter, the mean and standard deviation are provided in parenthesis. The bold numbers indicate a better result in comparison with the other approaches.

Parameter	Personal Zone Radius (m)	ROS Navigation	[34] Method	Proposed Approach
$d_{t}$ (m)		**87.33 (2.69)**	104.00 (7.94)	107.00 (7.55)
τ (s)		**11.80 (0.42)**	11.96 (0.65)	12.58 (0.71)
$d_{min}Person1$ (m)	1.6	0.56 (0.04)	1.24 (0.04)	**1.62 (0.01)**
$d_{min}Person2$ (m)	0.6	0.48 (0.04)	**1.10 (0.01)**	0.85 (0.04)
$d_{min}Person3$ (m)	0.9	0.52 (0.05)	**1.08 (0.12)**	1.11 (0.05)
Psi(Personal) (%)		12.49 (8.99)	5.75 (9.96)	**0 (0)**

**Table 3:** The questionnaire filled out by the participants of the experiment, highlighting in bold the changes between the approaches. CD (Completely Disagree), D (Disagree), N (Neither agree or disagree), A (Agree), CA (Completely Agree).

Item	[34] Method	Our Approach
“I have been comfortable with the presence of the robot.”	D	D
“The noise produced by the robot has bothered me.”	D	D
“The distance between the robot and me has been adequate.”	CD	**CA**
“The sudden movements of the robot have bothered me.”	D	D
“The robot has a human-like motion.”	N	N
“The robot behaves reliably.”	N	**A**

**Table 4:** T-student analysis applied to the results of Question 3 (“The distance between the robot and me has been adequate.”).

		[34] Method	Our Approach
Mean		2.33	3.83
Variance		2	1.21
Observations		18	18
Pearson Correlation	−0.76		
Hypothesized Mean Difference	0		
Variance of the Differences	5.56		
df	17		
t Stat	−2.7		
P (T ≤ t) two-tail	0.015		
